# Invasive Infections with *Nannizziopsis obscura* Species Complex in 9 Patients from West Africa, France, 2004–2020^1^

**DOI:** 10.3201/eid2609.200276

**Published:** 2020-09

**Authors:** Dea Garcia-Hermoso, Samia Hamane, Arnaud Fekkar, Arnaud Jabet, Blandine Denis, Martin Siguier, Guy Galeazzi, Elie Haddad, Sophie Brun, Valérie Vidal, Gilles Nevez, Rozenn Le Berre, Maud Gits-Muselli, Fanny Lanternier, Stéphane Bretagne

**Affiliations:** Institut Pasteur, Paris, France (D. Garcia-Hermoso, F. Lanternier, S. Bretagne);; Hôpitaux Lariboisière–Saint-Louis-Fernand Widal, Assistance Publique–Hôpitaux de Paris, Paris (S. Hamane, A. Jabet, B. Denis, M. Siguier, M. Gits-Muselli, S. Bretagne);; Groupe Hospitalier Pitié–Salpêtrière, Assistance Publique–Hôpitaux de Paris, Sorbonne Université, Paris (A. Fekkar);; Hôpital Max Fourestier, Nanterre, France (G. Galeazzi);; Centre Hospitalier Universitaire Hôtel-Dieu de France, Université de Saint-Joseph, Beirut, Lebanon (E. Haddad);; Hôpital Avicenne, Assistance Publique–Hôpitaux de Paris, Bobigny, France (S. Brun, V. Vidal);; Hôpital La Cavale Blanche, Centre Hospitalier Universitaire de Brest, Brest, France (G. Nevez, R. Le Berre);; Université Paris 13, Paris (S. Brun);; Université de Paris, Paris (M. Gits-Muselli, F. Lanternier, S. Bretagne)

**Keywords:** *Nannizziopsis*, *Onygenales*, invasive fungal infections, West Africa, emergence and immunosuppression, fungi, France

## Abstract

Nine new human invasive infections caused by the keratinophilic fungi *Nannizziopsis obscura* have been reported in France since 2004. The patients had variable clinical manifestations, had frequent dissemination, were mainly T-cell immunocompromised, and all originated from sub-Saharan West Africa. Before collection of the isolates, the etiologies of these infections were often misidentified, underscoring the extent of microscopic and cultural polymorphisms. All isolates but 1 had low MICs for the 8 antifungal drugs tested. When treated, patients received mainly azole therapy. Two of 7 patients with a known outcome died. We performed multilocus sequence analysis of *N. obscura* clinical strains and several strains of *Nannizziopsis* spp. isolated from reptiles. The human strains were clearly differentiated from the animal strains. *N. obscura* might be endemic to West Africa and responsible for undetected infections, which might become reactivated when immunosuppression occurs. *N. obscura* infection is probably underestimated because only sequencing enables proper identification.

## Abstract 2

Detection of these deep-seated infections suggests either the emergence of new fungal agents or improved means of identification.

*Nannizziopsis* spp. are described as keratinophilic ascomycetous fungi that cause dermal infections with frequently fatal outcomes in various reptiles (e.g., lizards, geckos, chameleons, iguanas, snakes, and crocodiles), mainly in captivity (*1*,*2*). *Nannizziopsis* spp. belong to the order of *Onygenales* and the recently described family of *Nannizziopsidaceae* (*1*). In humans, invasive *Nannizziopsis* spp. infection seems rare; only 5 cases have been reported to date (*1*,*3*–*7*). Such an observation suggests an actual rarity, a recent emergence because of modification in the ecoepidemiology (e.g., new populations at risk [*8*]), or previous underdiagnoses or misdiagnoses because of a lack of definite identification.

We describe 9 human cases of invasive fungal infection with *N. obscura* species complex identified in France during 2004–2020 (Table 1), along with the initial identification from the 5 reporting hospitals. Isolates were sent to France’s National Reference Center for Invasive Mycoses and Antifungals, where a polyphasic identification combining phenotypic features and molecular data was performed. Seven of the 9 cases were diagnosed after 2016.

**Table 1:** Clinical and laboratory characteristics of invasive infections with *Nannizziopsis obscura* in 9 patients from West Africa, France 2004–2020*

Patient no./ CNRMA strain no.	Year of diagnosis	Patient age, y/sex	Underlying risk factors	Country of birth/time since last travel in Africa	Clinical, radiologic, and biological findings	Direct examination	Serum β-D-glucan (Fungitell), pg/mL	Suspected identification at diagnosis	Treatment (duration)	Outcome
P1/4.1162	2004	49/M	AIDS	Mali/2 mo	Liver abscess†	Hyphae and arthroconidia	Not done	*Trichosporon* sp., *Chrysosporium* sp.	LAMB (unknown)	Lost to follow-up
P2/9.1232	2009	50/M	Heart transplant 2 mo earlier	Mali/NA	Fungemia,† cutaneous ulcers	Not done	Not done	*Chrysosporium* sp.	None	Death 2 d after diagnosis
P3/17.78	2016	58/F	Diabetes, Renal transplant (2017)	Mali/NA	Disseminated subcutaneous abscess† (legs, back) and pulmonary nodules	Septate, vesiculous mycelia, clavate sessile conidia	>500	*Trichophyton rubrum*	Posaconazole	No relapse after 1 y
P4/17.507	2017	62/M	Renal transplant (2009)	Guinea/1 y	Suppurated lesions (right ankle)† and lung micronodules, β-D-glucan+	Hyphae and arthroconidia	>500	*Trichosporon* sp.	Posaconazole	No relapse after 2 y
P5/18.682	2018	69/F	Mantle cell lymphoma (2017)	Guinea-Bissau/2 y	Pulmonary nodules, bronchial ulcerations and hyperchromic skin nodules,† β-D-glucan+	Hyphae and arthroconidia	>500	*Trichophyton rubrum*	Terbinafine, then voriconazole and LAMB	Death 6 wks after skin biopsy
P6/18.740	2018	28/F	Not known	Guinea/2 y	Mediastin (deep abscess)†	Hyphae	306	*Trichophyton rubrum*	Posaconazole, then voriconazole	Lost to follow-up
P7/19.38	2018	38/M	Renal transplant (2015)	Mali/1 y	Brain abscess†	Septate hyphae	>500	*Candida* sp.	LAMB and fluconazole, then LAMB and voriconazole	Alive 12 mo later
P8/19.607	2019	79/M	Renal transplant (2014)	Mali/3 y	Subcutaneous abscess (hand)† and pulmonary nodules, β-D-glucan+	Septate, vesiculous mycelia, clavate sessile conidia	255	*Trichophyton rubrum*	Itraconazole ongoing	Alive 4 mo later
P9/20.123	2019	65/M	Renal transplant (2018)	Mali/6 mo	Subcutaneous abscess (clavicle) and fistula with pus	Hyphae	>520	*Nannizziopsis* sp.	Voriconazole (ongoing)	Alive

*CNRMA, Centre National de Référence Mycoses Invasives et Antifongiques; LAMB, liposomal amphotericin B. †Original site of isolation (in cases when multiple sites were sampled).

## The Patients 

Patient 1 was 49-year-old HIV-positive man from Mali who was hospitalized for a liver abscess discovered in August 2004 during a stay in Mali. He was afebrile but had advanced AIDS (zero CD4 cell/mm^3^). A liver needle aspiration showed hyphae with arthroconidia. The first identified colonies were *Trichosporon* spp., based on a positive urease test and presence of arthroconidia. The patient was given liposomal amphotericin B and metronidazole. After 15 days, the patient returned to Mali for personal reasons; no follow-up was possible. At that time, the organism had been identified as *Chrysosporium* spp.

Patient 2 was a 50-year-old man who came from Mali to undergo heart transplantation in January 2009 after 9 months of hospitalization for cardiac insufficiency. After transplantation, the patient had cytomegalovirus reactivation and multivisceral failure. One month later, he had onset of bacterial mediastinitis. He was surgically treated and received wide-spectrum antibiotics but no antifungals. The immunosuppressive therapy consisted of prednisone (15 mg/d) and ciclosporine. A serum sample was negative for *Aspergillus* galactomannan. Two months later (just 2 days before the death of the patient), a blood culture was positive, and the isolate was identified as *Geotrichum* spp. or *Chrysosporium* spp.

Patient 3 was a 58-year-old woman with diabetes who was from Mali but had been living in France for 30 years. In 2017, she reported a 2-week history of asthenia and chest pain without fever. She had renal transplantation in 2016 and was receiving tacrolimus, mycophenolate, and prednisone (5 mg/d). A computed tomography (CT) scan revealed an irregular lung nodule (14 mm in diameter). She received amoxicillin/clavulanic acid. Three months later, she had an abscess of the left thigh and multiple nodular skin lesions on both legs. A new CT scan showed an enlargement of the pulmonary nodule. Direct examination of the skin and lung biopsies revealed septate and vesiculous hyphae, and the culture resembled *Trichophyton* spp., which was eventually confirmed as *N. obscura* upon sequencing. Serum β-D-glucan was strongly positive (>500 pg/mL, positivity threshold >80 pg/ml), and serum *Aspergillus* galactomannan antigen was repeatedly negative. A whole-body positron emission tomography (PET)–CT scan showed multiple clinically latent hypermetabolic lesions (in the nasal septum, left breast, and mediastinal nodes). Voriconazole was initiated, then switched to posaconazole after *N. obscura* identification. The dose of tacrolimus was reduced, and mycophenolate mofetil was replaced by azathioprine. At 6 months, a new PET-CT scan showed a residual hypermetabolic pulmonary lesion. Posaconazole was stopped after 8 months. No relapse had occurred as of 1 year later.

**Figure 1:**
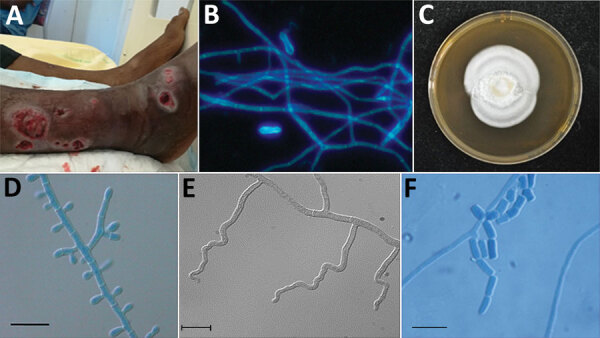
Features of *Nannizziopsis obscura* infections in patients from West Africa, France 2004–2020. A) Ulcerative lesions. B) Septate hyphae and arthroconidia on a calcofluor white direct examination (original magnification ×200). C) Macroscopic aspect on Sabouraud dextrose agar at 7 days. D) Septate conidiophore bearing clavate and sessile conidia. E) Undulate hyphae. F) Barrel-shaped arthroconidia. Scale bars indicate 10 µm.

Patient 4 was a 62-year-old man from Guinea who had been living in France for 12 years (recent trip to Guinea occurred »1 year before). He was hospitalized in July 2017 for several suppurated lesions on the right fibula (Figure 1, panel A) that were unresponsive to amoxicillin/clavulanic acid treatment. He had undergone renal transplantation in 2009 for hypertensive nephropathy and received mycophenolate mofetil, tacrolimus, and prednisone (5 mg/d). A CT scan confirmed tissue infiltration with small abscesses but showed no sign of bone involvement. Large-scale debridement was performed, and direct examination of infected tissues showed regular septate hyphae and arthroconidia (Figure 1, panel B). Yeast-like fungi appeared on Sabouraud–chloramphenicol–gentamycin slants. Microscopic examination showed arthroconidia, and the urease test was positive, suggesting the presence of *Trichosporon* spp. A whole-body PET-CT scan revealed asymptomatic hypermetabolic lesions in the contralateral leg and lung micronodules. Voriconazole was started when trichosporonosis was suspected and switched to posaconazole with the identification of *N. obscura*. Serum β-D-glucan was strongly positive (>500 pg/mL), whereas *Aspergillus* galactomannan antigen detection was negative. At 6 months, a PET-CT scan showed residual hypermetabolism around the right ankle. Onychomycosis of the right toe was noted, and a specimen was taken. Direct examination showed hyphae, but the culture was negative. Posaconazole was maintained for 2 years, with tacrolimus and prednisone (5 mg/d). A new PET-CT scan showed no hypermetabolic lesion.

Patient 5 was a 69-year-old woman from Guinea-Bissau who had been living in France for 10 years (her most recent trip to Guinea-Bissau occurred in 2016). She was admitted for the investigation of lung lesions. She had been treated for advanced mantle cell lymphoma in 2017, resulting in complete remission. In June 2018, a thoracic CT scan showed lymphadenopathy, lung nodules, and condensations treated with intravenous antibiotics and rituximab. In September 2018, disseminated nodular hyperchromic skin lesions appeared, and skin biopsies showed large septate hyphae with arthroconidia. A combination of voriconazole and liposomal amphotericin B was started for probable invasive mold infection. White mold colonies were observed after 5 days culture, and a presumptive identification of *Trichophyton* spp. was made. Terbinafine was added and but was exchanged for voriconazole and liposomal amphotericin B when *N. obscura* was identified. Thirty days after admission, magnetic resonance imaging of the central nervous system showed diffuse, embolic-looking ischemia. Viral PCRs were negative in cerebrospinal fluid. Serum β-D-glucan was strongly positive (>500 pg/mL), and serum galactomannan was negative. Liver biopsy confirmed adult T-cell lymphoma or leukemia associated with human T-cell lymphotropic virus type 1 positivity. The patient died 3 weeks after admission despite intensive care and antifungal treatment.

Patient 6 was a 27-year-old woman from Guinea who was breast-feeding. She had been living in France since September 2017 and was seen at a tuberculosis control center in October 2018. A chest radiograph showed a mediastinal mass, which was confirmed by a chest CT scan. The mass (7 × 5 × 5 cm) invaded the left upper lobe, and thickening of the anterior arch of the second left rib was observed. She had no notable medical history. Pathologic examination of the surgically resected mediastinal mass showed hyphae inside an inflammatory and fibrous reaction invading the thymus, the brachiocephalic veins, the left upper lobe, and the chest wall. Serum β-D-glucan was positive (306 pg/mL). A first presumptive identification on culture was of *Trichophyton* spp. Posaconazole was started, then switched with voriconazole when *N. obscura* was identified. A reduction in mass size (from 71 × 39 mm to 62 × 36 mm) was observed on a CT scan after 7 months of treatment. The investigations of Card9 and Stat1 mutations, 2 genes known to be responsible for higher susceptibility to invasive fungal infections (*9*), showed wild type genotypes. Follow-up after that point was not possible.

Patient 7 was a 38-year-old man from Mali who was hospitalized for visual disturbance, retroorbital pain, and vomiting in September 2018. He had been living in France since 1990, making regular visits to relatives in Mali. He had undergone a renal transplant in 2015 and was receiving mycophenolate mofetil, tacrolimus, and prednisone (5 mg/d). He had experienced an acute rejection in March 2018, which was treated by high-dose methylprednisone. A brain CT scan showed hypodense lesions with mass effect. Results of magnetic resonance imaging without injection supported the diagnosis of glioblastoma. A cerebral biopsy showed numerous branched hyphae. *Candida* spp. infection was suspected, and liposomal amphotericin B (5 mg/d) was started in combination with fluconazole IV (200 mg/d). The treatment resulted in an improvement of the visual disturbance, and a new magnetic resonance imaging result confirmed the reduction of the mass effect. Culture results were positive, and the final identification made was *Nannizziopsis* spp. Serum β-D-glucan was strongly positive (>500 pg/mL), and serum galactomannan was negative. On October 9, fluconazole was switched for voriconazole, and the liposomal amphotericin B was maintained. Meanwhile, the patient experienced a chronic rejection of the graft, and hemodialysis was restarted on November 23. He was alive as of 12 months later.

Patient 8 was a 79-year-old man from Mali who had been living in France since 1963 (his most recent trip to Mali occurred in 2016). In August 2019, he sought care for an ulcerative lesion of the fifth right finger that had been evolving for several months. He had undergone a renal transplant in 2014 and was receiving mycophenolate mofetil, tacrolimus, and prednisone (5 mg/d). A pulmonary nodule was observed in 2017 and was only surveyed. Because of a recent increase in size (from 9 to 13 cm in diameter), a PET-CT scan was performed in April 2019 and showed multiple hypermetabolic pulmonary, abdominal, and muscular (left thigh) nodules. The pulmonary nodule was surgically removed in June 2019, and a necrotic abscess with hyphae was observed by the pathologist (no culture was performed). The evolution was indolent, although the patient had lost 10 kg in 6 months. In August 2019, a skin biopsy showed hyphae, and the culture was identified as *N. obscura* upon sequencing. Serum β-D-glucan was positive (255 pg/mL), and serum galactomannan was negative. Itraconazole was started in August 2019. The patient had been seen in July 2018 because of the same ulcerative lesion of his right hand. A swab specimen yielded a mold colony identified as *Trichophyton rubrum*, which was considered not clinically relevant. The identification of the stored isolate yielded *N. obscura*, which confirmed that the infection had been ongoing for >1 year. The patient was well as of 4 months after starting azole therapy.

Patient 9 was a 65-year-old man from Mali who had been living in France for >20 years. In December 2019, he sought care for a mass in front of the left clavicle that had appeared 6 months earlier during a stay in Bamako, Mali. The patient had undergone a renal transplant in 2018 and was receiving cyclosporine, tacrolimus, and prednisone (5 mg/d). A CT scan showed bone lysis of the clavicle with a subcutaneous abscess. A biopsy was performed, and the pathologist reported inflammation with hyphae. Spontaneous fistula occurred, yielding pus. *Nannizziopsis* sp. was identified in the culture. A PET-CT showed hypermetabolism of the left clavicle, and the presternal region extended to the manubrium along with hypermetabolism of a pulmonary nodule of the lingula. Serum β-D-glucan results were positive (>520 pg/mL). Treatment with voriconazole was started and a reappraisal scheduled after 12 weeks.

## Materials and Methods

### Morphologic Identification and Antifungal-Susceptibility Testing

Ten clinical isolates (2 isolates for patient 8) were checked for purity and subsequently subcultured on potato dextrose agar (PDA) (BD Diagnostic Systems, https://www.bd.com; ttps://www.fishersci.fr/) and malt extract agar (MEA) 2% (Oxoid, http://www.oxoid.com) for 15 days at 30°C, 37°C, and 40°C to study fungal growth and sporulation. The type strain of *N. obscura* (isolate no. UAMH5875) was analyzed in parallel. Microscopic characteristics were examined on 5- to 7-day-old MEA slide cultures incubated at 30°C. Antifungal-susceptibility profiles were screened according to a slightly modified European Committee on Antimicrobial Susceptibility Testing procedure (*10*). All antifungal drugs were purchased from Alsachim (https://www.alsachim.com).

### Molecular Characterization and Phylogenetic Analysis

We performed DNA extraction and amplified fragments of the internal transcribed region (ITS), the D1–D2 region of the large subunit (LSU) ribosomal DNA, and the actin gene (*11*) (Appendix). We conducted a preliminary similarity searching using BLASTn (https://blast.ncbi.nlm.nih.gov) against curated fungal reference databases. We conducted multiple sequence alignments and single-gene phylogenies in MEGA7 (*12*). In addition to the clinical isolates and the type strain of *N*. *obscura*, we incorporated the corresponding sequences of *N*. *draconii*, *N*. *chlamydospora*, *N*. *guarroi*, *N*. *vriesii*, and *N*. *arthrosporioides* that are published in GenBank (Appendix). *N. hominis* was not included because of the lack of LSU and actin sequences in the public databases. Phylogenetic analysis was done with a neighbor-joining method by using MEGA7 and with the maximum-likelihood method by using PhyML 3.0 (*13*) subjected to smart model selection at the NGPhylogeny integrative web service (https://ngphylogeny.fr) (*14*).

### Ethics Considerations

We obtained approval from the Commission Nationale de l’Informatique et des Libertés, the national data-protection agency in France (approval no. 903395). This step ensured that the patients’ data were kept anonymous according to national regulations.

## Results

### Morphology

All clinical strains and the type strain grew well on PDA at 30°C and 37°C. No growth was observed at 40°C. Cultures on PDA and MEA at 30°C were white with low aerial mycelium and a velvety to powdery texture, rarely zonate, or heaped and with a yellowish coloring on the reverse (Figure 1, panel C). In general, microscopic observations showed the typical, although nonspecific, features of the genus *Nannizziopsis* (e.g., hyaline, septate, smooth-walled hyphae). All isolates produced sessile conidia and arthroconidia, and some produced short hyphal branches in a wavelike motion (undulate hyphae) (Figure 1, panels D–F) (*5*).

### Antifungal Susceptibility Testing

The MICs or minimal effective concentrations (MECs) of all 8 antifungals were low except for 1 strain. Median MICs were 0.25 mg/L (range 0.06–1 mg/L) for amphotericin B, 0.125 mg/L (range 0.014–4 mg/L) for itraconazole, 0.06 mg/L (range 0.03–2 mg/L) for voriconazole, 0.06 mg/L (range 0.014–2 mg/L) for posaconazole, 0.125 mg/L (range 0.06–2 mg/L) for isavuconazole, and 0.06 mg/L (range 0.014–0.5 mg/L) for terbinafine. Median MEC was 0.5 mg/L (range 0.25–1 mg/L) for caspofungin and 0.015 mg/L (range 0.015–0.06 mg/L) for micafungin.

### Molecular Characterization and Phylogenetic Analyses

Similarity comparisons in public databases showed that all isolates belong to the genus *Nannizziopsis* and had percentage identity ranges of 96.0%–99.8% (475 bp length) for LSU, 88.0%–99.0% (>700 bp length) for ITS, and 85.0%–98.7% (>500 bp length) for actin genes. *N. guarroi* (GenBank accession no. MH874904) had the highest number of hits for LSU, whereas *N. vriesii* (accession no. HF547893) had the highest number of hits for the actin gene. For ITS, the highest-scoring hits corresponded to a *Nannizziopsiaceae* strain (GenBank accession no. MF688808; 99%), followed by *Nannizziopsis* spp. (GenBank accession no. KY771169; 98.7%).

Multiple alignments for ITS2, LSU, and actin regions consisted of 283, 476, and 572 positions, of which 33 (11.6%), 31 (6.5%), and 128 (22.4%) were variable, respectively. The topologies observed on individual gene trees were very similar to those observed on a combined tree. The combined LSU-actin-ITS2 dataset of 1,331 positions had 192 (14.4%) of variable nucleotides.

**Figure 2:**
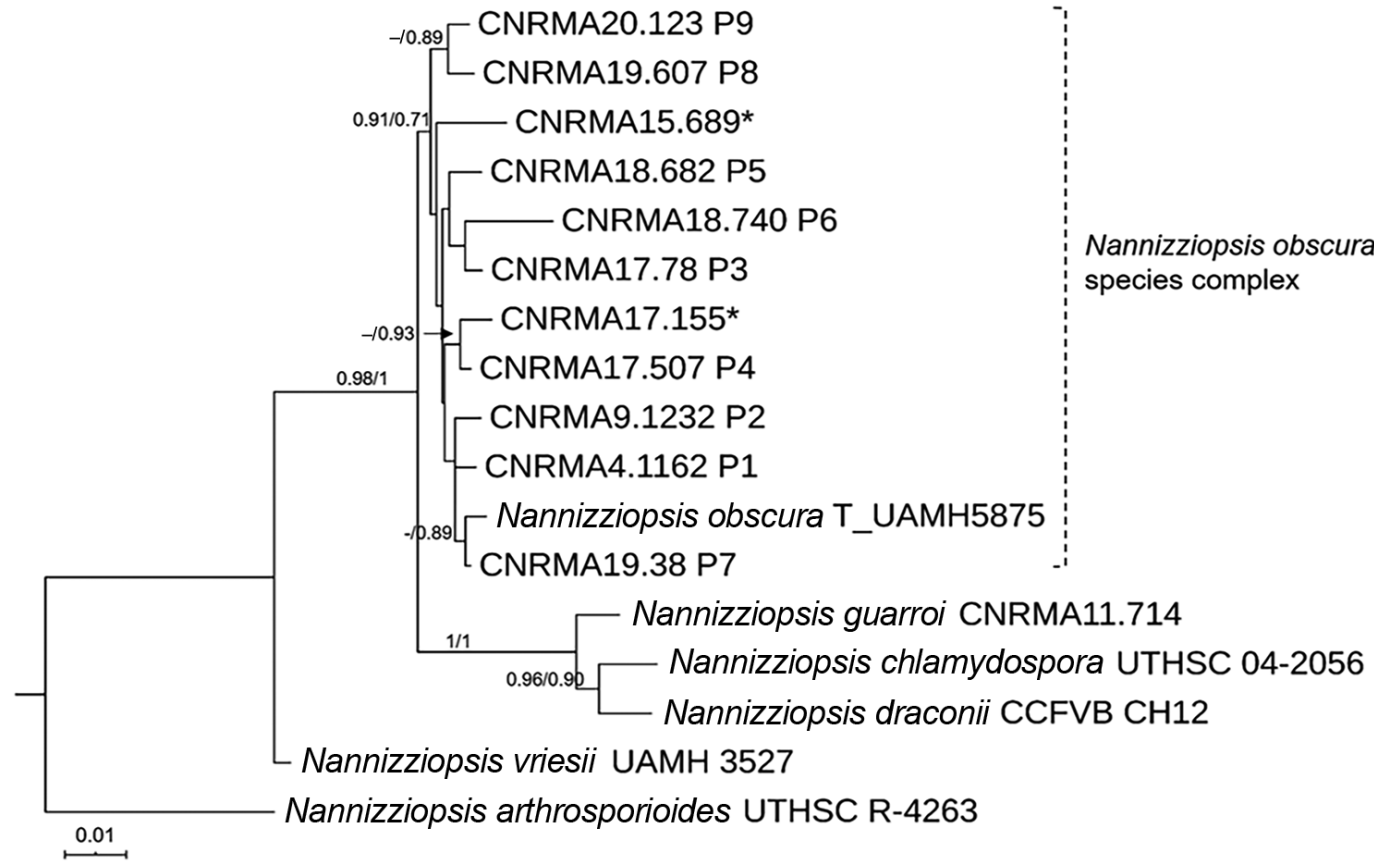
Maximum-likelihood tree obtained from combined large subunit ribosomal DNA, actin, and internal transcribed spacer 2 sequence data obtained from genomic analysis of *Nannizziopsis obscura* isolates from 9 patients from West Africa, France, 2004–2020, and reference sequences. Neighbor-joining bootstrap values or maximum-likelihood values are indicated on the branches. Support branch values <70% are not shown. Culture collection numbers appear next to sequences retrieved from GenBank, and type strains are indicated by a T after the species name. Patients from whom clinical isolates analyzed in this study were obtained are shown as P1–P9. The 2 isolates from patient 8 were morphologically and molecularly identical. Sequences marked with asterisks (*) refer to strains published by Nourrisson et al. (*6*). Scale bar indicates nucleotide substitutions per character.

The multilocus phylogenetic analysis revealed 2 main well-supported clades: 1 grouping all the clinical isolates, including the type strain of *N*. *obscura* and the named *N. obscura* species complex clade, and an additional clade assembling the 3 reptile *Nannizziopsis* species isolated from *Iguana iguana* (*N*. *guarroi*) and from bearded dragons, *Pogona vitticeps* (*N*. *draconii*, *N*. *chlamydospora*). *N. vriesii* and *N*. *arthrosporioides* were separated from the rest of the isolates and from each another (Figure 2).

## Discussion

We describe 9 new cases of proven invasive infection with the *N. obscura* species complex in France. The most frequent clinical localizations were subcutaneous tissues (6 patients) and lung nodules (6 patients) (Table 1). Eight of the 9 patients had T-cell immunosuppression associated mainly with the prevention of solid organ rejection. The constant feature was the sub-Saharan origin of all patients. The infecting agent was initially misidentified as *Geotrichum* spp., *Trichosporon* spp., and *Trichophyton* spp. in the 5 different participating hospitals (Table 1). Confusion of *Nannizziopsis* spp. with *Geotrichum* spp. (*3*,*5*), *Trichosporon* spp., or *Trichophyton* spp (*5*) are also common in the literature (Table 2). The most common finding upon direct examination at the microbiologic laboratories was the presence of nonspecific hyphae. On culture, *Nannizziopsis* spp. do not exhibit specific features (yeast-like or woolly aspect). Some characteristics of *Nannizziopsis* spp. are even shared with dermatophytes (e.g., cycloheximide tolerance and aleurioconidia). Dermatophytes can be involved in aggressive skin diseases, especially after renal transplantation (*15*). Moreover, empiric azole treatment might control the infection without a definite diagnosis. Because multilocus sequencing of the pathogen is often restricted to unusual localizations or therapeutic failures, a clear knowledge of the spectrum of *Nannizziopsis* spp. infections is lacking, which can explain, at least in part, the rarity of the cases reported.

**Table 2:** Clinical and laboratory characteristics of invasive infections with *Nannizziopsis obscura* as previously reported in the literature*

Year of diagnosis	Sex/age, y	Underlying risk factors	Country of birth/time since last travel in Africa	Clinical, radiologic, and biologic findings	Direct examination	Serum β-D-glucan (Fungitell)	Suspected identification at diagnosis	Treatment (duration)	Outcome	Ref
Before 1982 (reported in 1984)	M/24	HIV, osteomyelitis	Africa/7 y	Tibia (abscess)	Septate hyphae and budding yeasts	Not done	*Geotrichum* sp. and then *Chrysosporium* sp.	Amphotericin B (4 mo)	Persistent lytic area in the distal tibia after 2 y	(*3*)
2005	M/38	HIV	Nigeria (living in Germany)/NA	Brain (abscess, needle aspiration); lung (nodules)	Not available	Not done	*Chrysosporium* anamorph of *Nannizziopsis vriesii*	Voriconazole	Recovery without sequelae after 4 mo	(*4*)
2015	F/63	T-cell prolymphocytic leukemia (2014)	France/6 mo (Senegal)	Blood (blood culture, positive PCR on CSF, ascites fluid)	Septate hyphae, arthroconidia	Not done	*N. obscura*	Not treated	Dead before diagnosis	(*6*)
2015	M/34	Renal transplant (2008)	Gambia/3 mo	Back (paraspinal abscesses); lymph nodes (needle aspiration)	Aleurioconidia and arthroconidia in chains	Not done	*N. obscura*	Posaconazole	Recovery after 10 mo of azole therapy	(*7*)
2017	F/52	AIDS	Mali/resident	Brain (abscess), lung (nodule), β-D-glucan+	Not done	953 pg/mL	*N. obscura*	LAMB (1 mo), craniotomy, then voriconazole	Recovery but neurologic sequelae after 2 mo	(*6*)

*CSF, cerebrospinal fluid; LAMB, liposomal amphotericin B; Ref, reference.

The initial diagnosis also can be confused by the considerable diversity of the clinical manifestations. Some infections appeared as subacute; others were relatively indolent during periods of months or years. Records for these 9 patients and from the show that the underlying diseases are also diverse, although dominated by HIV infection before 2006 (3 patients) and solid organ transplantation (8 patients) after 2006; the most frequent clinical localizations were subcutaneous tissues (8 patients) and lung (7 patients) (Tables 1, 2). For some patients, the infection manifested as disseminated disease with brain abscess, lung nodules, or positive blood culture (Tables 1, 2). The association of serum β-D-glucan positivity and galactomannan negativity (7 and 6 patients tested in the 9-patient series, respectively) seems a useful adjunct, albeit unspecific. As a consequence, the suspicion of *Nannizziopsis* spp. infection cannot rely on a specific clinical manifestation and requires a tissue biopsy.

Our molecular study places all 10 *N*. *obscura* isolates (including 2 recovered from patient 8) into a well-supported phylogenetic lineage, separate from reptile isolates (Figure 2). Recent taxonomic revisions for the former *Chrysosporium* anamorph of *N. vriesii* complex resulted in the assignment of several species within the genus *Nannizziopsis* or within the 2 new genera of *Paranannizziopsis* and *Ophiodiomyces* (*1*,*5*). So far, only 2 species (*N*. *obscura* and *N*. *hominis*) have been definitely implicated in human pathology (*3*,*5*). The species *N*. *infrequens* was determined not to be responsible for an invasive infection and was disregarded by clinicians (*16*). Thus, *N*. *hominis* was reported in 3 patients before 2000, and *N*. *obscura* was reported in 4 patients after 2005 (*5*) and in 9 cases since then. *N*. *infrequens* and *N*. *hominis* exhibit good growth at 35°C, in contrast to the *Nannizziopsis* species implicated in reptile infections (*5*). Although our study clearly differentiates the human *N*. *obscura* isolates from our case series from the reptile isolates, the modest branch support value for the *N*. *obscura* clade (0.71 by maximum-likelihood method) suggests the possibility of potential new species. More taxa and additional gene sequences should be studied to investigate this hypothesis.

The issue of the portal of entry remains unclear. Subcutaneous nodules, ulcerative skin lesions, or both are frequently noted (e.g., in patients 2, 3, 4, 5, 8, and 9) (Table 1) and could have been the initial site of infection. Skin can be suspected because *Nannizziopsis* species are keratinophilic and cause extensive dermatitis with erosions and subsequent invasion of the subcutaneous structures in reptiles (*1*,*2*). When specifically investigated in this case series (i.e., patients 3 and 4) (Table 1), no *Nannizziopsis* organisms were recovered from skin or nail samples, even when patients had dermatomycoses or onyxis. On the other hand, the frequency of dissemination suggests inhalation as a possible route, with subcutaneous nodules as tissue localizations other than lung as a consequence of blood dissemination. Therefore, the infection scenario described for reptiles might not apply to humans.

We cannot provide firm recommendations for antifungal treatment because of the low number of patients in our study. The MICs show no intrinsic antifungal resistance. Azole therapy appears to be the first option, but nothing decides the choice between posaconazole or voriconazole except for the pharmacokinetics of each drug and its interactions with other medications. Decrease of immunosuppressive therapy might also contribute to improvement, and surgery can be a major part of treatment for some abscesses. However, as for many invasive fungal diseases, the final prognosis depends on that of the underlying disease.

**Figure 3:**
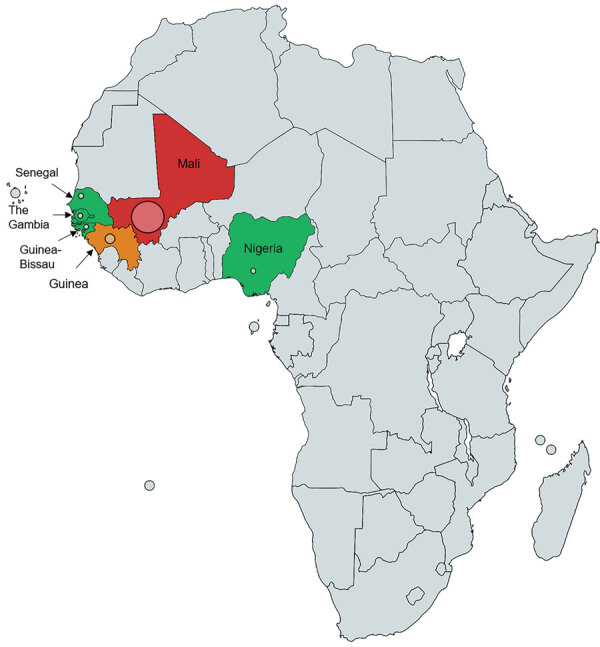
Geographic distribution of 13 patients infected by *Nannizziopsis obscura* in West Africa. The different colors represent the number of cases in each country: red for 7 cases, orange for 2 cases, and green for only 1 reported case. The diameter of the circle indicated for each country is proportional to the number of cases reported.

The main epidemiologic observation is the geographic origin of the patients. All came from sub-Saharan West Africa or Africa when national origin was reported (Tables 1, 2; Figure 3). The preferred migration routes explained by historical reasons—The Gambia to England route (*7*) and Mali, Senegal, and Guinea to France route—observed in our case-series and in the literature (*6*), might have introduced bias. However, France and England also have immigrants from other parts of Africa, and *Nannizziopsis* spp. infections are described only in patients from semiarid countries. For the previously reported patients from Nigeria (*4*,*5*), the exact origin was not reported, but Nigeria also covers semiarid tropical zones. In patients for whom the information was known, the delay between the last trip to Africa and the onset of symptoms varied between 2 months and 3 years (Table 1). Patients could carry latent forms of the fungus and have onset of an opportunistic infection when their immunity fails, as described for other fungi, such as *Cryptococcus neoformans* (*17*) and *Histoplasma* spp. or other endemic mycoses (*18*).

These 9 new cases and the previously reported cases (*1*,*3*–*7*) highlight the difficulties in identifying the *N. obscura* species complex. These fungal infections are likely underdiagnosed because of features shared with more common species, such as *Trichophyton* spp. or *Trichosporon* spp. Matrix-assisted laser desorption/ionization time-of-flight mass spectrometry should accelerate the process of identifying organisms and updating public databases, such as the online mass spectrometry platform (https://msi.happy-dev.fr), which now includes a *N. obscura* profile. Up to now, these deep infections seemed to involve T-cell immunosuppressed patients with frequent dissemination or multifocus localizations. These molds are probably endemic in sub-Saharan Africa, but their precise geographic repartition and natural ecology remain to be established. Environmental studies would be necessary to further investigate the natural ecology of these molds, as has been done recently for the emerging *Emergomyces africanus* (*19*).

Appendix Additional information about invasive infections with *Nannizziopsis obscura* species complex in 9 patients from West Africa, France, 2004–2020.
