# Supplementary material for: Invasive Infections with Nannizziopsis obscura Species Complex in 9 Patients from West Africa, France, 2004–2020
Source: Emerg Infect Dis. 2020 Sep;26(9):2022–30. doi: 10.3201/eid2609.200276 (PMC7454062; doi:10.3201/eid2609.200276)
Supplement: Appendix — Additional information about invasive infections with Nannizziopsis obscura species complex in 9 patients from West Africa, France, 2004–2020. [file 20-0276-Techapp-s1.pdf]

Article DOI: https://doi.org/10.3201/eid2609.200276

Invasive Infections with *Nannizziopsis obscura* Species Complex in 9 Patients from West Africa, France, 2004–2020

Appendix

Materials and Methods

Molecular Characterization and Phylogenetic Analysis

For the genomic DNA extraction ≈100 mg of mycelium from a 5 day-old PDA culture at 30°C was suspended in 700 µL of ATL lysing solution (Qiagen, Germantown, MD, USA) containing 500 µL of ceramic beads, and disrupted on a MAGNA Lyser (Roche Diagnostics, Mannheim, Germany) instrument (three runs of 30s at 7,000 rpm). DNA was then purified using NucleoMag Plant (Macherey-Nagel, Düren, Germany) and the semi-automated KingFisher Flex magnetic particle processor (Thermo Fisher Scientific, Vantaa, Finland). Sequencing of PCR ITS, LSU and actin products was performed at Eurofins sequencing facility by using the cycle sequencing technology (dideoxy chain termination / cycle sequencing) on ABI 3730XL sequencing machines (Applied Biosystems). Consensus sequences were determined using the Sequencher version 5.4.6 sequence analysis software (Gene Codes Corporation, Ann Arbor, MI USA). Preliminary sequence similarity searching (BLASTn) was performed against curated fungal reference databases available at the online MycoBank database (http://www.mycobank.org/) and the NCBI GenBank (http://www.ncbi.nlm.nih.gov). Multiple sequence alignments were carried out using MAFFT v.7.308 with Geneious v11.0.5 with default settings. Single gene phylogenies for all three loci and estimation of the best substitution models were inferred using were inferred using MEGA7. The concatenated alignment was submitted to Neighbor joining (NJ) analysis using MEGA7 (*1*) and to Maximum likelihood (ML) analysis in the web server NGPhylogeny.fr. For NJ analyses of the combined dataset, the substitution model applied was Tajima-Nei method with a gamma distribution. Robustness of the branches was assessed by bootstrap analysis with 1000 replicates. For ML tree construction, the selected model

by Smart Model Selection (SMS) was GTR general time-reversible model according Akaike Information Criterion (AIC) test criterion. Internal branch reliability was assessed using the aLRT test (SH-Like) (*2*).

Appendix Table. GenBank accession numbers for the *Nannizziopsis obscura* species complex sequences reported in this study and those available in the literature

Patient no. (strain no. or reference)	GenBank accession numbers		
	ITS2	LSU	Actin
P1 (CNRMA4.1162)	MN982937	MN982951	MT024770
P2 (CNRMA 9.1232)	MN982938	MN982952	MT024771
P3 (CNRMA17.78)	MN982939	MN982953	MT024772
P4 (CNRMA17.507)	MN982940	MN982954	MT024773
P5 (CNRMA18.682)	MN982941	MN982955	MT024774
P6 (CNRMA18.740)	MN982942	MN982956	MT024775
P7 (CNRMA19.38)	MN982943	MN982957	MT024776
P8 (CNRMA19.607)	MN982944	MN982958	MT024777
P9 (CNRMA20.123)	MT345076	MT341813	MT350285
Stilwell, 1984 (*3*)	NR111878	NA	NA
Steiniger 2005 (*4*)	HF547869	HF547853	HF547877
Baggott 2017 (*5*)	NA	NA	NA
Nourrisson 2018 (*6*)	KY771168	NA	NA
Nourrisson 2018 (*6*)	KY771169	NA	NA

References

1. Kumar S, Stecher G, Tamura K. MEGA7: Molecular Evolutionary Genetics Analysis version 7.0 for bigger datasets. Mol Biol Evol. 2016;33:1870–4. PubMed https://doi.org/10.1093/molbev/msw054

2. Anisimova M, Gascuel O. Approximate likelihood-ratio test for branches: a fast, accurate, and powerful alternative. Syst Biol. 2006;55:539–52. PubMed https://doi.org/10.1080/10635150600755453

3. Stillwell WT, Rubin BD, Axelrod JL. *Chrysosporium*, a new causative agent in osteomyelitis. A case report. Clin Orthop Relat Res. 1984; (184):190–2. PubMed

4. Steininger C, van Lunzen J, Sobottka I, Rohde H, Horstkotte MA, Stellbrink H-J. Mycotic brain abscess caused by opportunistic reptile pathogen. Emerg Infect Dis. 2005;11:349–50. PubMed https://doi.org/10.3201/eid1102.040915

5. Baggott A, McGann H, Barton R, Ratner J. Disseminated *Nannizziopsis obscura* infection in a renal transplant patient—the first reported case. Med Mycol Case Rep. 2017;17:20–4. PubMed https://doi.org/10.1016/j.mmcr.2017.06.002

6. Nourrisson C, Vidal-Roux M, Cayot S, Jacomet C, Bothorel C, Ledoux-Pilon A, et al. Invasive infections caused by *Nannizziopsis* spp. molds in immunocompromised patients. Emerg Infect Dis. 2018;24:549–52. PubMed https://doi.org/10.3201/eid2403.170772
